# A multicomponent quasi-experimental ergonomic interventional study: long-term parallel four-groups interventions

**DOI:** 10.1186/s12891-023-06220-4

**Published:** 2023-02-09

**Authors:** Reza Esmaeili, Mahnaz Shakerian, Sayed Vahid Esmaeili, Mahdi Jalali, Amin Babaei Pouya, Azim Karimi

**Affiliations:** 1 Student Research Committee, Department of Occupational Health and Safety Engineering, School of Health, Isfahan University of Medical Sciences, Isfahan, Iran; 2 Department of Occupational Health and Safety Engineering, School of Health, Isfahan University of Medical Sciences, Isfahan, Iran; 3 Department of Occupational Health and Safety Engineering, School of Public Health and Safety, Shahid Beheshti University of Medical Sciences, Tehran, Iran; 4 Department of Occupational Health Engineering, School of Health, Neyshabur University of Medical Sciences, Neyshabur, Iran; 5 Department of Occupational Health and Safety Engineering, School of Health, Ardabil University of Medical Sciences, Ardabil, Iran; 6 Student Research Committee, Department of Occupational Health Engineering, School of Health, Isfahan University of Medical Sciences, Isfahan, Iran

**Keywords:** Intervention study, Musculoskeletal disorder, Ergonomics training, Workstation modification, Foundry workers

## Abstract

**Background:**

Musculoskeletal disorders (MSDs) are known as one of the main problems affecting the health of industrial workers and can lead to lost working days, functional disability of workers and wasting the financial resources of an organization. Therefore, the present study aimed to evaluating the effect of ergonomic interventions on reducing MSDs and improving working posture in the in a foundry industry workers.

**Methods:**

A field multicomponent cross-interventional study was conducted on workers working in a foundry industry. In this study, 117 male workers were divided into 4 groups, including a control group, a group with specialized ergonomics training, a group with workstation intervention, and a group simultaneously undergoing training and workstation intervention. All 4 groups were evaluated during a period of baseline, 6 and 12-months follow- up. The Cornell Musculoskeletal Discomfort Questionnaire (CMDQ) and direct observations of working postures by using the Quick Exposure Check (QEC) method were used.

**Results:**

The results showed that the implemented interventions in the shoulder/arm, back and stress level were effective and the difference in the final score was significant among different groups (*P*-value > 0.05). In addition, the interventions led to a significant decrease in the QEC scores and musculoskeletal symptom scores in the neck, shoulder, lower back, knee, and lower leg regions among different groups (*P*-value > 0.05).

**Conclusion:**

The results showed that workstation modification and training and workstation intervention simultaneously had a greater effect on MSDs and improving working posture compared to training alone.

## Background

In today’s competitive world, the optimal use of company’s resources to reduce industrial costs is great of importance. Therefore, it is necessary to prepare the work environments more comfortable and effective for the workforce [1]. Work-related musculoskeletal disorders (WMSDs) are considered as a major issue for workers and organizations worldwide [2]. WMSDs lead to losing working days, disabilities or functional limitations of workers; moreover, they also cause reducing the quality of life and wasting money [3]. Ergonomics interventions are concerned with making the workplace environment as efficient, safe, and comfortable as possible. Effective application of ergonomics interventions to work system design can result in a positive balance between worker characteristics and task demands. This balanced-effort can enhance the workers productivity and also provide them safety, physical and mental well-being and job satisfaction [4]. Recently, numerous studies have reported that WMSDs are related to both the physical and psychological perceived job demands in the workplace [5–7]. WMSDs associated with working posture (physical conditions) result from conditions where workers experience discomfort or pain in one or different body parts, such as neck, shoulder, back, elbow, hand, hip and knee, pain in the joints, and/or tingling, and swelling [8]. Work posture can be defined as the orientation of body parts in a specific work area while the worker is performing a task [9]. Working posture is determined by the characteristics of worker, the design of workstations, and the processes being undertaken. Dimensions, spatial position, orientation, and design of workstation must suit the physical needs of the workers, so that they can perform the designated task in a safe working posture. In general, workers can perform their jobs at their workplaces either while standing or sitting, and using a combination of both positions [10].

Ergonomics intervention studies intend to reduce the WMSDs such as the studies in which researchers follow or arrange changes in working conditions to determine the effects of modifications on the risk factors and/or health [11, 12]. Several controlled workplace studies reported the positive effect of ergonomic intervention on WMSDs symptoms with adjustable workstations [13], a sit–stand height adjustable workstation [14], increased frequency of work breaks [15], ergonomics training, physical exercises, and improved lighting conditions [16]. Moreover, it has been reported that a modified workstation can reduce ergonomic problems, enhance operator performance [17], and elevate employee performance, which will lead to higher quality in manufacturing [18].

The metal foundry is the most efficient and economical procedure to produce metals used in a broad range of industries. The foundry is a primary industry in a manufacturing process in which a liquid material is poured into a designed model to make a cast [19]. Foundry process activities have a high potential to cause WMSDs; because the majority of the tasks are performed manually. Research on the physical load of the workers in the foundry industry demonstrated that the activities of breaking and debarring impose a high physiological demand, once the difference of the heart rate between resting and performing those activities exceeds the acceptable physiological limits of the working load [20].

The study conducted in a foundry plant showed that nearly 84% of male and 76% of female workers reported WMSDs [21]. The results of another study carried out in Chinese foundries illustrated that the most affected areas after 12 months of work are the back (29.2%), shoulders (10.5%), and hands and wrists (6.2%) [22]. Another study about the physical load of workers in Brazilian foundries showed 75.2% of workers reported some WMSDs symptoms in the past 12 months, 53.3% reported symptoms the last seven days, and 38.5% reported already taking time off due to this problem [20].

The foundry industry consists of several parts and various risk factors which can exacerbate WMSDs. The mentioned risk factors include reaching, bending, lifting heavy objects, continuous force, working with vibrating equipment, and repetitive motion. Therefore, implementing safety and health policies to protect workers seems necessary. The lack of attention of the foundry industry management to safety and health, the use of only traditional and inefficient methods to provide safety for working conditions, and spending little time and money for planning and executing a developed safety program are the most important factors that have made the working environment in Pakistan one of the most accident prone and hazardous working environments. On the basis of the above-mentioned background issues and the recommendations made by other studies [23] for beneficial ergonomics interventions, the current research was conducted to investigate the effect of workstation modifications and ergonomics training on decreasing of WMSDs and working posture improvement and also to achieve following objectives:

- Ergonomics evaluation of workers and determination and identification of ergonomics problems in personnel working in a foundry industry
- Investigating the effect of training on improving the ergonomic position and reducing MSDs of workers
- Investigating the effect of workstation modifications on improving ergonomic condition and reducing MSDs of personnel
- Investigating the simultaneous effect of training and workstation modifications on improving the ergonomic position and reducing MSDs of personnel

## Materials and methods

### Study design and setting

This 12-month period quasi-experimental interventional study was conducted at a foundry plant in 2022 in Isfahan, Iran. The study period was between March 2021 and April 2022. The company employed a total of 180 workers at the time of the study; approximately 135 of them were working in four different production units. Of the production unit employees, 15 workers were excluded from the study, due to night shift activities and the lack of access to them, and 3 other workers were excluded due to their unwillingness to participate in the study. Therefore, the final study population included 117 workers of the production unit. The company's official working hours were stated as being 7 AM to 4 PM; however, the workers were often required to work until 6 PM in the most working days, due to high workload and the need for the timely delivery of orders. Initial observations revealed sufficient scope for ergonomic design interventions in this foundry. Before the study began, the purpose of the study was fully explained to the participants, they were assured that their personal information would remain confidential, and they were also told that they could withdraw from the study at any stage. The inclusion criteria of this study included as follows: a minimum of one year’s experience at this foundry; and no apparent physical or mental problems (based on self-reporting).

### Participants

This field-interventional study consisted of collecting data at baseline and the introduction of a modified workstation and ergonomics training with 12-month follow-up. To ensure that each group contained participants with similar workload requirements and job descriptions, the workers were not randomly assigned to the study groups. Also, with regard to ensure that the study participants had no influence on each other, in terms of the measured outcomes and practices that could potentially affect the study results, the recruited workers were assigned accordingly and fairly by management. The groups were physically and geographically separated and the 4 groups were selected from different production units. Participants were assigned to one of the following four study groups:

1. Control group (no intervention);
2. Modified workstation-only (WS-only) intervention group;
3. Ergonomics training-only (T-only) intervention group;
4. Modified workstation + ergonomics training (WS + T) intervention group.

**Fig. 1:**
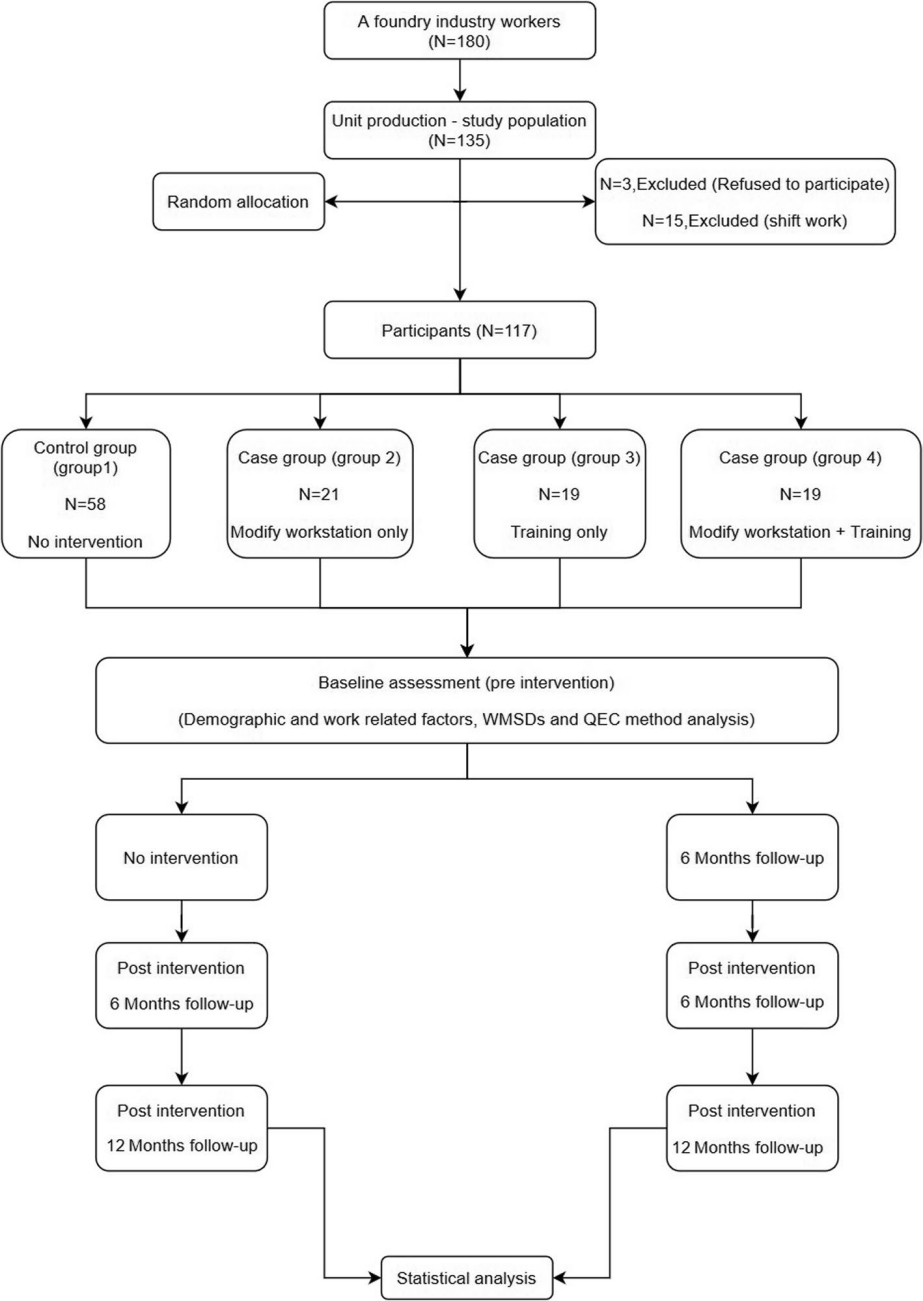
Flow diagram of the study design

The four groups were studied before (baseline) and after (6- and 12-month follow-ups) the intervention. The data were collected through an anonymous questionnaire, which consisted of three parts and covered the following items: demographic factors (including age, height, weight, education level, and involvement in regular sport/physical activities each week), work-related musculoskeletal disorders (WMSDS, using the standardized Cornell Musculoskeletal Discomfort Questionnaires [CMDQ]) [24]. Direct observations of the participants during their work were also performed, using the quick exposure check (QEC) method [25]. A flow diagram of the study design is shown in Fig. 1.

### The foundry company process

Foundry is actually the art of shaping metals and alloys by melting and pouring molten material into a container called a mold and then cooling and solidifies it in to the shape of the mold container [26]. This method is the oldest known process for shaping metals. This process can be very diverse and different depending on its technology [27]. In the present study, the sand mold casting process was used. The present study has been carried out on a foundry industry with a sand mold. Casting parts in this industry range from light (weight of 3 kg) to heavy (weight of 3 tons). But most of the casting parts were in the range of less than 100 kg. Therefore, due to speeding up the working process, manual handling of parts was unavoidable in many cases. The most important issue regarding the ergonomic condition of the work environment, however, was the grinding of small and medium parts, which the operator had to grind on the floor in a very inappropriate posture (Bending the knee, bending the back, holding the parts with the tip of the foot and using the grinding stone at a relatively far distance from the body due to safety precautions and preventing the grinding stone from touching the body). For the above-mentioned reasons, the number of MSDs in annual medical examinations and workers MSDs complaints was high. Therefore, it was decided to investigate this issue in a longitudinal interventional study.

### Steering committee (SC) development

As previously mentioned, the main objective of the present study was to investigating the effect of workstation modifications, ergonomics training and their simultaneous effect on musculoskeletal symptoms and working Postures. To achieve this goal, a steering committee (SC) was formed consisting of 14 experts including 3 ergonomics university expert, 2 experts from the HSE unit of the foundry company, the foundry CEO, a production manager, a technical manager, 3 production supervisors, workers' representative and 2 occupational health inspectors of the regional health center. The SC was responsible for evaluating and identifying ergonomics problems, feasibility and providing control measures, implementing control measures, monitoring the continuity of activity and evaluating the effectiveness of control measures. Accordingly, SC had a direct role in selecting interventions including workstation modification and designing ergonomic training programs.

### Ergonomic interventions

#### Modifying workstation

Based on the direct observations of workers tasks and activities, the recognized risk factors using ILO (International Labor Organization) checkpoint [28] and the results of the evaluation of workers postures several technical and practical solutions have been recommended by action and research groups to provide a better fit based on workers needs and those were applied with the support of the SC.

In this study, interventions related to workstations modification were performed for both WS-Only and WS + T groups and included the following:

- Workstations redesign through height adjustment
- Preparation of specialized tables with different heights for locating parts of different sizes
- Providing ergonomic chairs according to the body dimensions of the users
- Fixing parts for grinding operations using fixtures

**Fig. 2:**
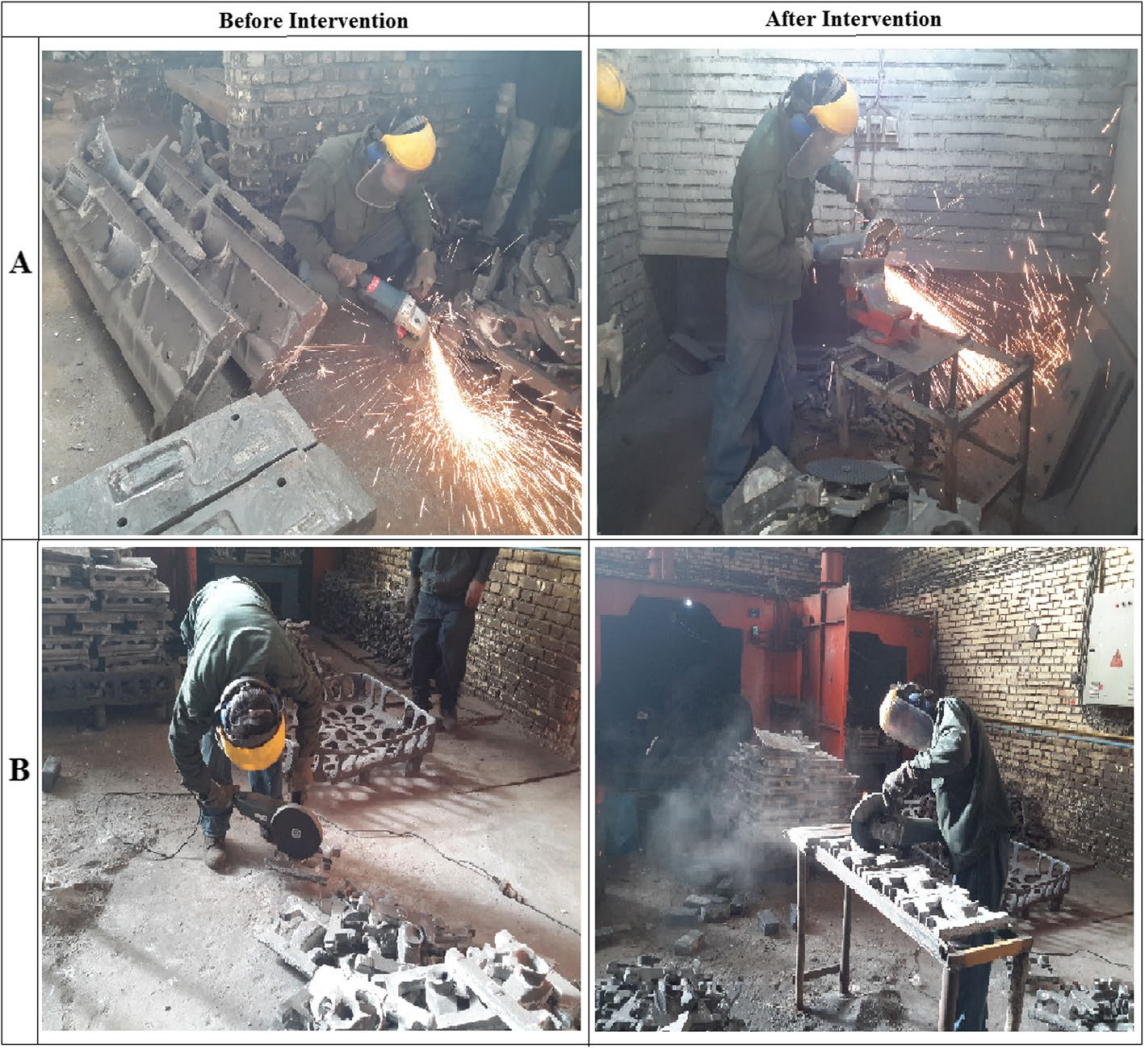
Some workstations before and after intervention. **A** Grinding worker for large parts; **B** Grinding worker for small parts

For example, in grinding workers as one of critical activities, based on pre-intervention results of posture analysis using QEC three regions of their body including back, shoulder/arm and wrist/hand had the most score than other regions of their body. Accordingly, an ergonomic table was designed for improvement of their working posture. The existing fixture used for grinding workers is shown in Fig. 2. Generally, workstation modifications were made in such a way that the level of discomfort experienced by the workers was as low as possible.

#### Ergonomics training

Ergonomic training program was set according to workers' demands. Therefore, all necessary courses were determined by SC members and approved by them. As mentioned above, results of foundry process task analysis revealed that foundry workers had frequent awkward postures during their work. Hence, the main objective of this program was to improve ergonomics awareness in all workers—from OHS officials to front-line workers—about the consequences of adopting inappropriate and awkward postures during their work. The training program consisted of four sessions during one month. Each week featured a 90-min session, 60 min of highly specialized workplace ergonomics, and 30 min of participants’ questions and answers. In addition, general ergonomics tips were provided, such as healthy lifestyles, healthy diet, exercise, ideal weight, driving ergonomics, and sleeping ergonomics. This program lasted two months, during which eight training sessions were held for the participants. The titles of the implemented ergonomic training program consist of: Work-related musculoskeletal disorders, ergonomic postures, ergonomics of working with a grinder. The sessions were conducted by the HSE manager in the studied foundry industry, and they included instructional video clips, photos, power point presentations, handouts, and practical training by the instructor.

At the end of each training session, the participants received a training brochure. A written test was administered before the start of each new training session based on the brochure and the training materials, and a prize was awarded to the participants with the highest scores (as a motivational item).

### Instruments and outcome measures

#### Ergonomic evaluation

In order to evaluate the ergonomic risk factors, the quick exposure check (QEC) was used in the current study as a rapid method for evaluating the exposure of workers to risk factors associated with MSDs [29]. The reliability, validity, and usability of the QEC were evaluated in two stages with 206 participants [25]. The QEC can be used to assess a wide range of tasks, including carrying loads and repetitious, dynamic, and sit-and-stand tasks [30]. The benefit of this method compared with the other similar methods is that it considers psychosocial stop-here risk factors in addition to the typical physical risk factors [31]. QEC is a good way to evaluate ergonomic interventions in the workplace. Therefore, a re-evaluation of an interventional strategy can be made immediately after changes in the work environment [25]. The QEC evaluates four regions of the body for repetitive postures and movements, i.e., the back, shoulders and arms, wrists and hands, and neck. The final score (i.e., low, moderate, high, or very high) is calculated based on the exposure time, the combination of risk factors encountered, and the occupational stress occurred in each region of the body (33). After the final score has been determined, ergonomic interventions can be planned for the workers. Workers were given a form designed specifically for the study, and they were asked to list the problems and physical stresses they encountered. In the current study, the independent variable was the participant's ergonomic status, including clumsy posture measurable by QEC. The awkward postures found in the casting process were distinguished by several observational events conducted by the research team. Postures were found as long as workers' working hours and work-related psychological stress of activities involving activities with awkward pustures were comprehensively recorded for the majority of participants. We recommend observing 20 to 30 cycles of a particular activity first. It is worth noting that the noteable feature of QEC to be used is cooperation and interaction found between the observers and the participants while working in all different evaluation steps, initially the priority to be investigated is selected. The researchers then carefully observed the worker's posture while asking the participants specific questions about their posture and various body movements. Participants finally answer the other required questions on the checklist. All evaluations were done both before and after the implementation of the interventions by constant persons.

#### Musculoskeletal Disorders (MSDs)

To identify any MSDs, the participants completed the Persian version of the Cornell Musculoskeletal Discomfort Questionnaires (CMDQ). This instrument which was developed by Allen et al. in 1999 [32] and was used in the United States and other countries, could be considered as a valid and reliable way of evaluating ergonomic efforts [33]. It is also well-known as a valuable tool for assessing the extent of MSDs and is focused on three features in the timeframe of a week: the frequency of discomfort (never, 1–2 times last week, 3–4 times last week, once every day, and several times every day), the severity of discomfort (slightly uncomfortable, moderately uncomfortable, and very uncomfortable), and the impact of discomfort on work capacity (not at all, slightly interfered, and substantially interfered). The data are recorded on a map depicting 12 regions of the body (neck, shoulders, upper back, upper arms, lower back, forearms, wrists, buttocks, foot, thighs, knees, and lower back), or 20 parts of the body in total.

By study intent, the CMDQ was used to assess the presence of musculoskeletal pain, discomfort, or impairment, the intensity of the discomfort, and its potential impact on the worker's ability to work during work. To obtain more accurate results, participants were instructed on how to complete the questionnaire before starting the survey. To meet the study intent, the research team participated in a goal workshop in which participants completed questionnaires.

### Statistical analysis

Data analysis was performed using SPSS version 22. The four groups were compared at baseline and after intervention (6-month follow-up and 12-month follow-up) using McNemar (ANOVA) and chi-square analyses for continuous and categorical variables, respectively. Cochran's Q test was used to evaluate the prevalence of WMSDs, whereas repeated-measures analysis of variance (ANOVA) was used to assess working posture and CMDQ scores before and after the implementation of the intervention program. For all analyses, *p* < 0.05 was considered statistically significant. After analysis, modified workstations were developed based on the results of posture evaluation and observation of occupations and activities. The modifications were made in accordance with the ergonomic principles and in collaboration with the manager and manager of production units to ensure postural tensions were as low as possible when working.

## Results

### Baseline information

Of the 135 initial participants, 117 completed the study, 58 in Group control (no intervention received) and others in 3 groups that were received the interventions. The demographic information and job details of the 4 groups (control, WS-only, T-only and WS + T) are presented in Table 1. The mean (standard deviation [SD]) age of the 4 groups include: control, WS-only, T-only and WS + T was 31.7 (8.25) years, 30.33 (7.12) years, 31.05 (8.40) years and 34.15 (10.50) years, respectively. Most of the participants did not have college degree (control = %93.1, WS-only = %90.5, T-only = %100 and WS + T = %89.5) and the mean (SD) duration of their work experience of the 4 groups was 6.74 (5.17) years, 6.28 (3.78) years, 6.36 (5.14) years and 7.68 (5.53) years, respectively. There were no significant differences between the four studied groups in terms of demographic and job details.

**Table 1:** Baseline characteristics of participants (*n* = 117)

Demographic characteristics	Groups				*P* value (type of statistical analysis)
	Control (*n* = 58) *Mean* ± *SD/n (%)*	WS-only (*n* = 21) *Mean* ± *SD/ n (%)*	T-only (*n* = 19) *Mean* ± *SD/ n (%)*	WS + T (*n* = 19) *Mean* ± *SD/ n (%)*	
**Age (years)**	31.7 ± *8.25*	30.33 ± *7.12*	31.05 ± *8.40*	34.15 ± *10.50*	0.654 (ANOVA)
**BMI (Kg/m2)**	24.35 ± *4.61*	23.10 ± *1.55*	24.21 ± *1.27*	25.47 ± *7.76*	0.766 (ANOVA)
**Education level**					
**Under Diploma**	44 (75.9)	15 (71.4)	14 (73.7)	14 (73.7)	0.848 (chi-square)
**Diploma**	10 (17.2)	4 (19)	5 (26.3)	3 (15.8)	
**bachelor**	4 (6.9)	2 (9.5)	0	2 (10.5)	
**Smoking**					
**Yes**	23(39.7)	6 (28.6)	6 (31.6)	7 (36.8)	0.80 (chi-square)
**No**	35 (60.3)	15 (71.4)	13 (68.4)	12 (63.2)	
**Exercise**					
**Yes**	10 (17.2)	7 (33.3)	3 (15.8)	3 (15.8)	0.382 (chi-square)
**No**	48 (82.8)	14 (66.7)	16 (84.2)	16 (84.2)	
**Work experience (years)**	6.74 ± *5.17*	6.28 ± *3.78*	6.36 ± *5.14*	7.68 ± *5.53*	0.813 (ANOVA)
**Prevalence of WMSDs**					
**Neck**	12 (20.7)	4 (19)	4 (21.1)	4 (21.1)	0.998 (chi-square)
**Shoulder**	15 (25.9)	5 (23.8)	5 (26.3)	5 (26.3)	0.997 (chi-square)
**Upper back**	6 (10.3)	2 (9.5)	3 (15.8)	2 (10.5)	0.763 (chi-square)
**Upper arm**	12 (20.7)	2 (9.5)	3 (15.8)	2 (10.5)	0.331 (chi-square)
**Lower back**	21 (36.2)	8 (31.8)	7 (36.8)	7 (36.8)	0.999 (chi-square)
**Forearm**	15 (25.9)	5 (23.8)	5 (26.3)	6 (31.6)	0.952 (chi-square)
**Wrist**	8 (13.8)	3 (14.3)	3 (15.8)	4 (21.1)	0.896 (chi-square)
**Thigh**	7 (12.1)	2 (9.5)	2 (10.5)	1 (5.3)	0.865 (chi-square)
**Knee**	18 (31)	6 (28.6)	6 (31.6)	7 (36.8)	0.935 (chi-square)
**Lower leg**	13 (22.4)	6 (28.6)	5 (26.3)	5 (26.3)	0.943 (chi-square)
**Foot**	11 (19)	4 (19)	4 (21.1)	4 (21.1)	0.955 (chi-square)
**Score Exposure Level**					
**Back**	29.82 ± 5.58	29.61 ± 5.78	29.78 ± 5.73	30.21 ± 5.88	0.990 (ANOVA)
**Shoulder/arm**	29.93 ± 4.23	29.52 ± 3.94	29.47 ± 4.15	29.47 ± 4.15	0.954 (ANOVA)
**Wrist/hand**	30.34 ± 3.39	30.66 ± 3.48	29.89 ± 3.49	30.31 ± 3.48	0.917 (ANOVA)
**Neck**	15.75 ± 1.59	15.61 ± 1.62	15.78 ± 1.61	15.57 ± 1.57	0.961 (ANOVA)
**Vibration**	4.31 ± *3.97*	4.04 ± *3.98*	4.36 ± *4.05*	4.36 ± *4.05*	0.993 (ANOVA)
**Work pace**	7.27 ± 2.39	7.33 ± 2.41	7.42 ± 2.38	7.15 ± 2.47	0.995 (ANOVA)
**Stress**	8.18 ± 4.08	7.85 ± 4.15	7.73 ± 4.35	8.78 ± 3.88	0.858 (ANOVA)

### Exposure levels (based on QEC analysis) at pre and post intervention phases

Table 2 shows QEC scores for the pre, post 1 and post 2 interventions of the studied population. The mean QEC score of all part of assess at baseline among the 4 groups, respectively, corresponded to an action level of high risky, which indicated that most operators needed an investigation and modifications in their working habits soon. At pre, post 1 and post2 intervention, there were significant differences in the back in 2 groups (WS-only and WS + T) (*p* < 0.001), shoulder/arm in 2 groups (WS-only and WS + T) (*p* < 0.05) and stress in 2 groups (T-only and WS + T) (*p* < 0.05). The QEC scores were not significantly different at pre, post 1 and post-intervention 2 in the other parts and other groups of scores (*p* > 0.05).

**Table 2:** Distribution of QEC scores [mean (SD)] for the studied participants (*n* = 117)

Body region	Group	QEC final Score			F	*P*-value
		Pre-intervention	Post-intervention (1)	Post-intervention (2)		
**Back**	Control	29.82 (5.58)	28.82 (5.58)	29.58 (5.59)	2.38	0.128
	**WS-only**	**29.61 (5.78)**	**24.61 (3.55)**	**25.41(3.55)**	**8.75**	**0.001**
	T-only	29.78 (5.73)	27.73 (4.99)	27.43 (4.99)	2.44	0.101
	**WS + T**	**30.21 (5.88)**	**24.73 (2.99)**	**24.13 (2.99)**	**12.70**	**0.002**
**Shoulder/arm**	Control	29.93 (4.23)	30.20 (4.09)	30.44 (4.15)	3.64	0.531
	**WS-only**	**29.52 (3.94)**	**26.38 (1.20)**	**25.87 (1.43)**	**11.22**	**0.03**
	T-only	29.47 (4.15)	28.94 (3.67)	28.94 (3.67)	3.25	0.141
	**WS + T**	**29.47 (4.15)**	**26.21 (0.91)**	**25.21 (1.12)**	**10.13**	**0.047**
**Wrist/hand**	Control	30.34 (3.39)	30.48 (3.37)	30.24 (3.43)	1.49	0.231
	WS-only	30.66 (3.48)	30.47 (3.15)	30.28(3.53)	0.417	0.626
	T-only	29.89 (3.49)	29.89 (3.49)	29.68 (3.35)	1.00	0.378
	WS + T	30.31 (3.48)	29.89 (3.29)	30.00 (3.33)	0.468	0.550
**Neck**	Control	15.75 (1.59)	15.55 (1.54)	15.41 (1.49)	3.12	0.515
	WS-only	15.61 (1.62)	14.57 (2.01)	14.28 (2.21)	1.00	0.544
	T-only	15.78 (1.61)	15.78 (1.61)	15.47 (2.09)	2.01	0.651
	WS + T	15.57 (1.57)	14.63 (2.49)	14.63 (2.49)	1.07	0.426
**Work pace**	Control	7.27 (2.39)	7.18 (2.42)	7.18 (2.42)	1.00	0.322
	WS-only	7.33 (2.41)	7.09 (2.48)	7.80 (2.18)	2.5	0.105
	T-only	7.42 (2.38)	6.89 (2.53)	7.42 (2.38)	1.00	0.378
	WS + T	7.15 (2.47)	7.11 (2.47)	7.5 (2.47)	1.00	0.364
**Stress**	Control	8.18 (4.08)	8.65 (4.04)	8.53 (3.92)	1.88	0.169
	WS-only	7.85 (4.15)	6.95 (3.24)	7.19 (3.20)	2.53	0.114
	**T-only**	**7.73 (4.35)**	**6.84 (3.97)**	**6.57 (3.99)**	**3.67**	**0.035**
	**WS + T**	**8.78 (3.88)**	**5.95 (2.47)**	**6.12 (2.38)**	**4.41**	**0.001**
**Vibration**	Control	4.31 (3.97)	4.21 (3.91)	4.41 (3.83)	1.05	0.987
	WS-only	4.04 (3.98)	3.99 (3.78)	4.11 (3.94)	2.11	0.996
	T-only	4.36 (4.05)	4.42 (4.15)	3.96 (3.95)	1.00	0.898
	WS + T	4.36 (4.05)	3.99 (4.25)	4.33 (4.15)	3.12	0.976

**Significant difference (from repeated measures ANOVA analysis) between three time points**

### Musculoskeletal symptom scores

The most commonly WMSDs among the participants in four studied groups at baseline were the lower back (36.2% control, 31.8% WS-only, 36.8% T-only and 36.8% WS + T), knees (31% control, 28.6% WS-only, 31.6% T-only and 36.8% WS + T) and shoulders (25.9% control, 23.8% WS-only, 26.3% T-only and 26.3% WS + T). No significant difference was found between the 4 groups in terms of prevalence of WMSDs at baseline (Table 1). Table 3 shows the pre, post1 and post 2 intervention scores of CMDQ questionnaire of the studied population. As can be seen, significant differences were found between the score of neck (*p* < 0.05) in T-only and WS + T groups, shoulder (*p* < 0.05) in WS-only, T-only and WS + T groups, lower back (*p* < 0.05) in WS-only and WS + T groups and knee (*p* < 0.05) in WS-only and WS + T groups and lower leg (*p* < 0.05) in WS + T group, before and after the interventions. Also, no significant difference was found between the 4 groups in other body parts between before and after interventions. The CMDQ score among 4 groups and all body parts was not significantly different between the 2 interventions (e.g.post-intervention 1 and post-intervention 2).

**Table 3:** score of CMDQ questionnaire (*n* = 117)

Body part	Control (*n* = 58) *Mean* ± *SD*				WS-only (*n* = 21) *Mean* ± *SD*				T-only (*n* = 19) *Mean* ± *SD*				WS + T (*n* = 19) *Mean* ± *SD*			
	pre	Post 1	Post 2	*P*-value	pre	Post 1	Post 2	*P*-value	pre	Post 1	Post 2	*P*-value	pre	Post 1	Post 2	*P*-value
**Neck**	1.87 ± *6.13*	2.1 ± *6.44*	2.1 ± *6.23*	0.487	1.79 ± *2.85*	1.02 ± *1.72*	1.23 ± *1.56*	0.115	**2.15 ± *6.40***	**1.00 ± *1.56***	**0.9 ± *1.62***	**0.012**	**2.7 ± *7.14***	**1.33 ± *0.86***	**0.91 ± *0.22***	**0.033**
**Shoulder**	3.84 ± *9.07*	3.6 ± 9.01	3.9 ± 4.39	0.945	**4.14 ± *9.32***	**3.0 ± 9.55**	**2.4 ± 1.33**	**0.047**	**4.57 ± *9.72***	**0.63 ± 1.83**	**0.1 ± *0*.15**	**0.036**	**4 ± *8.52***	**0.1 ± *0*.31**	**0.1 ± 0.10**	**0.011**
**Upper back**	1.27 ± *5.91*	0.8 ± 3.70	1.1 ± 5.04	0.280	3.00 ± *9.58*	2.7 ± 6.58	2.6 ± 6.06	0.329	4.68 ± *12.84*	3.5 ± 3.83	2.9 ± 6.17	0.135	1.42 ± *6.19*	0.9 ± *0*.22	1.0 ± *0*.22	0.331
**Upper arm**	2.98 ± *7.10*	2.7 ± 6.5	2.5 ± 6.8	0.574	3.14 ± *6.81*	2.7 ± 4.04	2.3 ± 3.74	0.125	2.15 ± *5.71*	2.3 ± 5.37	2.1 ± 7.37	0.418	1.31 ± *6.04*	1.6 ± 0.22	0.9	0.113
**Lower back**	8.44 ± *14.31*	8.4 ± 14.1	11. ± 21.4	0.280	**11.28 ± *17.06***	**3.4 ± 8.18**	**3.8 ± 9.59**	**0.001**	8.89 ± *15.20*	6.7 ± 8.42	5.1 ± 8.49	0.075	**10.47 ± *15.73***	**3.8 ± 9.33**	**4.7 ± 11.4**	**0.001**
**Forearm**	5.81 ± *11.32*	5.12 ± *12.24*	4.99 ± *11.23*	0.428	5.21 ± *12.01*	3.9 ± 2.50	3.0 ± 2.12	0.244	4.88 ± *10.12*	3.3 ± 5.55	4.1 ± 6.12	0.325	5.11 ± *11.02*	4.17 ± *9.02*	5.81 ± *12.02*	0.376
**Wrist**	3.24 ± *9.90*	4.21 ± *6.21*	3.84 ± *7.42*	0.345	4.71 ± *11.94*	3.75 ± *8.35*	4.19 ± *6.85*	0.654	5.68 ± *13.81*	4.24 ± *8.94*	3.98 ± *7.56*	0.458	4.10 ± *11.56*	4.01 ± *6.56*	4.68 ± *7.12*	0.568
**Thigh**	2.31 ± *7.31*	1.87 ± *6.18*	1.87 ± *6.18*	0.377	3 ± *9.58*	2.61 ± *8.10*	2.61 ± *8.10*	0.167	1.84 ± *6.36*	1.05 ± *0.22*	1.05 ± *0.22*	0.220	1.89 ± *8.25*	1.05 ± *0.12*	1.12 ± *0.31*	0.338
**Knee**	5.9 ± 12.4	5.7 ± *11.2*	5.5 ± *11.7*	0.723	**6.5 ± 13.5**	**3.3 ± *8.45***	**3.3 ± *8.14***	**0.035**	6.3 ± *10.2*	5.6 ± *11.6*	5.7 ± *9.62*	0.231	**6.4 ± *10.7***	**1.0 ± *2.54***	**1.2 ± *2.56***	**0.019**
**Lower leg**	2.84 ± 6.94	3.51 ± *9.31*	3.36 ± *8.86*	0.119	3.76 ± 10.74	3.51 ± *9.31*	3.38 ± *7.77*	0.944	3.21 ± *10.76*	2.8 ± *2.54*	2.8 ± *2.54*	0.375	**1.02 ± *2.18***	**0.8 ± *2.54***	**0.52 ± *1.82***	**0.039**
**Foot**	2.8 ± 6.14	2.1 ± *5.74*	2.0 ± *4.78*	0.752	3.0 ± 8.16	3.1 ± *7.15*	2.8 ± *8.45*	0.842	3.4 ± *7.13*	4.9 ± *9.25*	3.0 ± *6.45*	0.873	4.1 ± *9.42*	5.0 ± *13.5*	5.9 ± *15.8*	0.975

**Significant difference (from repeated measures ANOVA) between three time points**

**Fig. 3:**
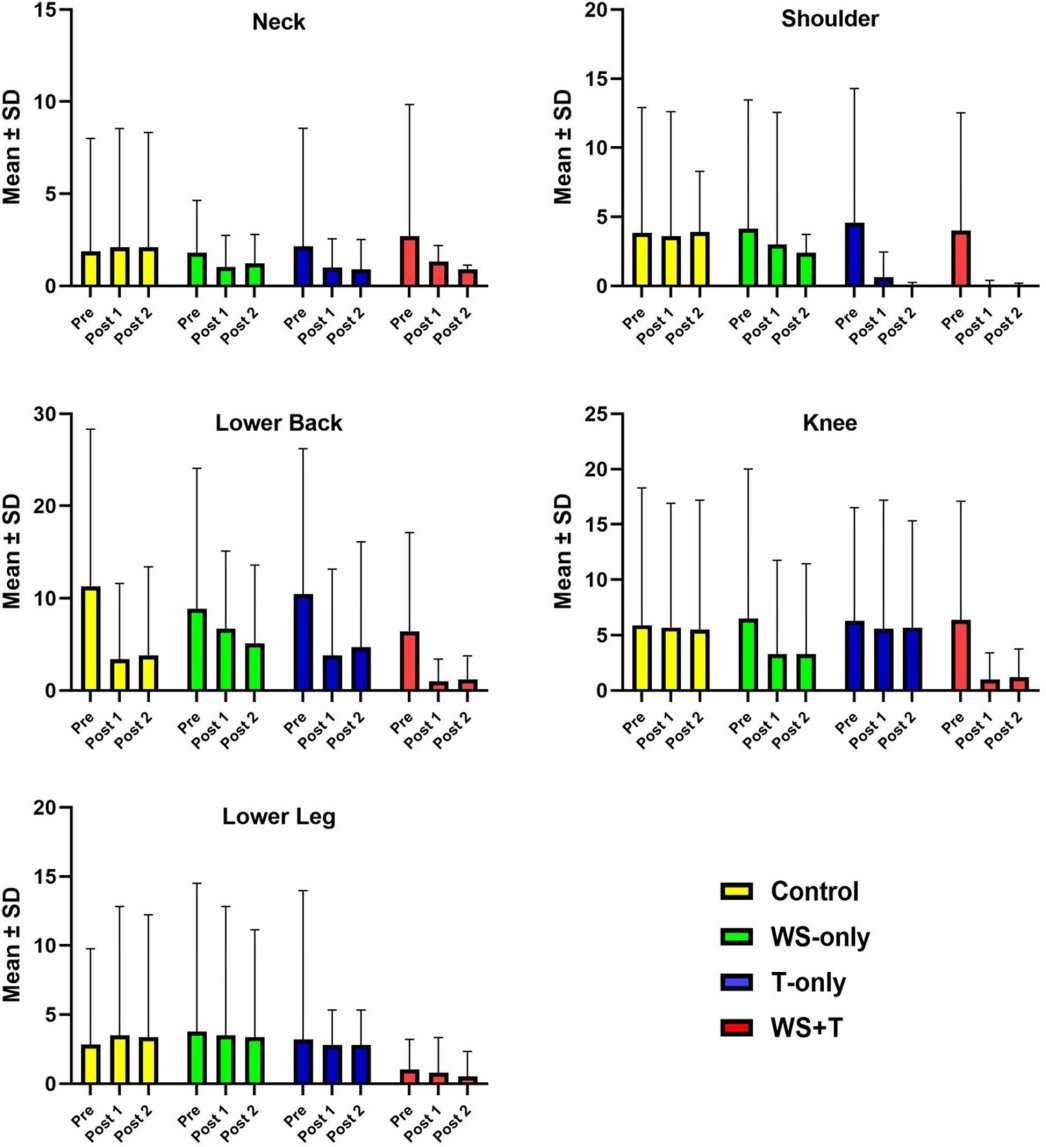
Comparison of the final CMDQ scores in the body regions that were most affected by the multicomponent interventions

Figure 3 illustrates the changes of the final CMDQ score in the body regions where the interventions had significant effects on this score. Accordingly, the neck chart shows that the changes in the final CMDQ score were significant in the T-only group and the WS + T group. According to shoulder region chart, the changes in the final CMDQ score were significant in all three intervention groups. The three other regions (Lower back, Knee, Lower leg) chart shows that the changes in WS-only and WS + T groups were significant. Finally, it can be interpreted that in foundry workers, the effect of workstation modification on the final CMDQ score was greater than the effect of training.

The results of investigation of the association between the mean scores of discomforts in various body parts (CMDQ), and the total points of risk assessment techniques (QEC) via Pearson regression analysis have been displayed in Table 4. Accordingly, the mean discomfort scores for the neck, lower back, wrists, and Shoulder were significantly association with mean posture assessment scores for the neck, back, Wrist/hand, and Shoulder/arm amongst the participants (*p* < 0.005).

**Table 4:** Pearson regression analysis between the prevalence of discomfort in different body parts, and final postural assessment scores

Scores based on CMDQ results	Scores based on QEC results					
	Back	Shoulder/arm	Wrist/hand	Neck	Work pace	Stress
**Neck**	R = 0.12	R = 0.56	R = 0.05	R = 0.95	R = 0.34	R = 0.63
	P = 0.34	P = 0.004	P = 0.001	P = 0.001	P = 0.03	P = 0.001
**Shoulder**	R = 0.36	R = 0.87	R = 0.06	R = 0.54	R = 0.12	R = 0.76
	P = 0.24	P = 0.001	P = 0.42	P = 0.03	P = 0.07	P = 0.02
**Upper back**	R = 0.66	R = 0.34	R = 0.14	R = 0.56	R = 0.25	R = 0.49
	P = 0.03	P = 0.06	P = 0.06	P = 0.02	P = 0.12	P = 0.001
**Upper arm**	R = 0.03	R = 0.89	R = 0.34	R = 0.07	R = 0.26	R = 0.78
	P = 0.51	P = 0.001	P = 0.42	P = 0.31	P = 0.54	P = 0.26
**Lower back**	R = 0.96	R = 0.26	R = 0.01	R = 0.34	R = 0.76	R = 0.68
	P = 0.001	P = 0.87	P = 0.86	P = 0.05	P = 0.02	P = 0.001
**Forearm**	R = 0.02	R = 0.12	R = 0.41	R = 0.21	R = 0.01	R = 0.03
	P = 0.34	P = 0.64	P = 0.35	P = 0.53	P = 0.35	P = 0.62
**Wrist**	R = 0.07	R = 0.31	R = 0.72	R = 0.02	R = 0.84	R = 0.67
	P = 0.82	P = 0.43	P = 0.04	P = 0.09	P = 0.001	P = 0.06
**Thigh**	R = 0.31	R = 0.24	R = 0.01	R = 0.16	R = 0.15	R = 0.03
	P = 0.87	P = 0.94	P = 0.76	P = 0.53	P = 0.34	P = 0.38
**Knee**	R = 0.41	R = 0.05	R = 0.18	R = 0.08	R = 0.38	R = 0.06
	P = 0.86	P = 0.61	P = 0.94	P = 0.31	P = 0.01	P = 0.64
**Lower leg**	R = 0.26	R = 0.25	R = 0.33	R = 0.19	R = 0.03	R = 0.21
	P = 0.35	P = 0.66	P = 0.05	P = 0.43	P = 0.56	P = 0.07
**Foot**	R = 0.06	R = 0.22	R = 0.51	R = 0.09	R = 0.09	R = 0.08
	P = 0.16	P = 0.73	P = 0.11	P = 0.31	P = 0.07	P = 0.54

## Discussion

Despite the high prevalence of MSDs and its symptoms in foundry workers, little is known about the effectiveness of intervention study programs aimed at mitigating such disorders in this workforce population. The present quasi-experimental study examined the effects of a longitudinal field workplace ergonomics intervention. This study formalized a multicomponent intervention program including three approaches of workstation modification, ergonomics training, and their simultaneous effect in a foundry industry and reduced physical ergonomics risk factors in three groups with one control group. Then, the results of evaluating each group were compared with others. Investigation of the impact of interventions in four separated groups can be considered as strength of the current work. The main findings of the study included the following:

The QEC method score, which was used to evaluate the level of exposure to risk factors related to MSDs, was significant in the back (in WS-only and WS + T groups), shoulder/arm (in WS-only and WS + T groups) and stress (in T-only and WS + T groups) areas between the pre and post interventions times (*P*-value > 0.05). The changes of QEC score in these areas were completely aligned with the interventions. Also, the changes in score related to the CMDQ questionnaire, which was used to evaluate MSDs, were measured in the body region of neck (in T-only and WS + T groups), shoulder (in WS-only and WS + T groups), lower back (in WS-only and WS + T groups), knee (in WS-only and WS + T groups) and lower leg (only in in the WS + T group) during three times (pre and post interventions) was significant (*P*-value > 0.05). These changes of CMDQ score were also in line with the interventions.

In this study, no significant relationship was found between WMSDs and QEC scores in the control group before and after the intervention. This finding is in line with the results of the study conducted by Bazazan et al. [34] conducted on control room operators in a petrochemical plant in which no association was found between WMSDs and RULA score. Our findings indicated considerable improvement in working postures (particularly in the back and shoulder/arm) and reducing in the prevalence of WMSDs (particularly in the neck, shoulder, lower back, knee and lower leg) after the intervention, which confirms the effectiveness of the implemented interventions. In a similar study on ergonomic interventions in the foundry industry, conducted by Susihono and Adiatmika, ergonomic interventions to reduce the risk of MSDs were examined [35]. The effect of ergonomic interventions in their study leads to a decrease in musculoskeletal complaints by 25.27%, a decrease in boredom by 25.01% and an increase in job satisfaction by 38.46%. Also, in another study by Colim [36] et al. in the packaging industry the implemented interventions on reducing MSDs were assessed. In the latest mentioned study, the researchers used RULA and Norodic methods to evaluate ergonomics and showed that waist torsion poses a lot of risk for workers through which to reduce these disorders, intervention robots were recommended to be applied. In another study, Owen et al. [37] showed that after using a five year ergonomics program, the rate of back, back and shoulder injuries were significantly reduced. The study of Kiroly et al. [38] in investigating the effect of ergonomics intervention on reducing the number of inappropriate physical postures and the prevalence of MSDs in female physicians, showed that after ergonomics interventions, 64% of awkward postures were corrected. Also, the ergonomics intervention that Choobineh et al. [39] performed in the embossing workstation of traditional workshops showed that by designing the embossing table, the working conditions were significantly improved and the priority level of corrective action was decreased from 3 before the intervention to 2 after that. The modified workstation corrected about 57% of the work postures.

The present research findings also showed that the workstation modification could improve work postures (in the back and shoulder/arm) and reduce the prevalence of WMSDs (in the shoulder, lower back, and knee). These results are in line with the interventional study carried out in the office workers who received workstation modification or new ergonomic equipment (e.g., chairs, forearm support) for improving workstation a reported significant reductions in WMSDs symptoms and improving working postures [40]. In addition, the obtained findings from furniture manufacturing industry study suggested that ergonomically redesigned workstation was an effective intervention program to reduce awkward postures in the trunk postures and trunk kinematics required to perform the requisite tasks [41].

Additionally, the results of this study illustrated training could be reducing prevalence of WMSDs (in the neck and shoulder) but training alone could not be improving working posture, just there was a significant difference only in stress control (QEC scores). These findings are consistent with those of Ketola et al. [42] presentation that trained groups in office ergonomics demonstrated less WMSDs than the reference group. In another similar study, Bohr et al. [43] found that those who received ergonomics training reported less pain/discomfort and work stress following the intervention than those who did not receive training. But in some studies, it has been reported that office ergonomics training significantly improves work postures [44, 45]. This disagreement may be due to the differences observed in the occupations examined. Casting industry tasks are heavy and work postures for the most part depend on workstation design.

The study of Abareshi et al. [46], shows the effect of training in reducing MSDs. This study shows that, before intervention in both the experimental and control groups, there were no significant differences among the average protection motivation theory constructs, productivity and QEC scores (*p* > 0.05). Along with, after training intervention, there was a significant increase in these factors within the investigated group apart from the perceived response costs and efficacy. Their study also shows that ergonomics training based on the protection motivation theory is effective in reducing MSDs risk factors and the promotion of knowledge of the subject which can increase productivity. These results were consistent with the results of our study. Kee [47] examined the use of ergonomics interventions and ergonomics training programs for nurses and concluded that these programs can significantly reduce back and lower back pain. So, the workstation modification was found more effective on participants than training in reducing the prevalence of WMSDs and improving work postures. As shown in this research, the simultaneous interventions of workstation modification and training have had a greater effect on reducing the prevalence of WMSDs and improving work postures.

In previous studies, in order to evaluate the impact of ergonomics interventions, the number of the percentage of reported disorders before and after the interventions were used as a measure of the effectiveness of the interventions [48, 49]. But in fact, sometimes the implemented interventions cannot cause the complete improvement of disorders, but it causes less pain intensity, less impact of pain on work and less frequency of feeling pain. In such a situation, an effective ergonomics intervention may be carried out, but the number of disorders before and after the implementation of the intervention does not show a significant difference and causes the wrong conclusion to be interpreted from the intervention. In order to prevent such cases and to have a more suitable criterion, it is suggested to use the scores of the CMDQ as a criterion to measure the effectiveness of interventions. The scores of this questionnaire provide a more accurate interpretation of the state of disorders due to the fact that in cases, where disorders do not recover completely, the interpretation of pain quality will be an important parameter for judgment.

The present study findings indicated that between QEC scores and CMDQ scores existed a positive correlation and, QEC served as a superior technique for predicting musculoskeletal problems. On the same basis, the study of Eyvazlou et al. [50], which was conducted in order to evaluate the musculoskeletal disorders of dentists, also showed a positive relationship between QEC scores and CMDQ results. In our study, with regard to the findings of postural evaluation, it was found that effective interventional strategies revolved around ergonomically redesigned work stations and improved working environments. Therefore, further research regarding WMSDs will effectively contribute to the awareness and reduction of musculoskeletal discomforts worldwide.

The results of this study should be considered in the context of its limitations, consist of: First, the improvements in outcome variables evaluated in this study were based on ergonomic intervention, which was conducted among a relatively small group of male employees. Thus, care should be taken in to account these findings to other ergonomic interventions and population groups. Future researches using larger sample sizes and different occupational groups may be required for the purposes of validation and generalizability. There also may be possible limitations with regard to the accuracy and reliability of self-reported symptom questionnaire and observational risk assessment tool. Therefore, further researches evaluating the outcome variables in a more objective manner (e.g., muscle activity and fatigue as well as video-based motion and electro goniometric measurements for musculoskeletal risk assessment) are also recommended.

## Conclusion

Due to the adverse effects of ergonomics problems on productivity and employee health, engineering and training interventions can be an effective step in reducing these problems. Performing engineering and training interventions in this study were able to reduce the level of MSDs and improve the working condition of employees and reduce MSDs in industry. Based on the results of the present study, the simultaneous use of posture evaluation methods can provide a more appropriate view of the current situation and the effectiveness of the interventions performed by the researcher. In this study, the simultaneous use of QEC and CMDQ methods gives a complete picture of the effect of interventions in reducing MSDs among workers and also these interventions will reduce employee stress and naturally increase workers' productivity.

## Data Availability

Due to the request of the participants in the study and the protection of their privacy, we are exempt from disclosing their personal information publicly. The datasets used and analyzed during the current study are available from the corresponding author on reasonable request.
